# Hepatitis C virus nonstructural protein 5A perturbs lipid metabolism by modulating AMPK/SREBP-1c signaling

**DOI:** 10.1186/s12944-019-1136-y

**Published:** 2019-11-04

**Authors:** Ziyu Meng, Qiang Liu, Fujun Sun, Ling Qiao

**Affiliations:** 1 NHC Key Laboratory of Hormones and Development, Tianjin Key Laboratory of Metabolic Diseases, Tianjin Medical University Chu Hsien-I Memorial Hospital & Tianjin Institute of Endocrinology, Tianjin, 300134 China; 2 Vaccine and Infectious Disease Organization-International Vaccine Centre (VIDO-InterVac), School of Public Health Vaccinology and Immunotherapeutics, Department of Veterinary Microbiology, University of Saskatchewan, S7N5E3, Saskatoon, Canada

**Keywords:** Hepatitis C virus, Nonstructural protein 5A, Hepatic steatosis, AMP-activated protein kinase, Sterol regulatory element-binding protein-1c

## Abstract

**Background:**

Steatosis is an important clinical manifestation associated with chronic hepatitis C virus (HCV) infection. AMP-activated protein kinase (AMPK), a major mediator of lipid metabolism, regulates HCV-associated hepatic steatosis, but the underlying mechanisms remain obscure. Here we investigated the mechanism of HCV nonstructural protein 5A (NS5A)-induced lipid accumulation by the AMPK/SREBP-1c pathway.

**Methods:**

We generated model mice by injecting recombinant lentiviral particles expressing the NS5A protein (genotype 3a) via the tail vein. The serum levels of alanine aminotransferase (ALT), free fatty acids (FFAs) and triglycerides (TG) were examined. H&E and Oil Red O staining were used to examine lipid droplets. Immunohistochemistry staining, quantitative real-time PCR and Western blotting were used to determine the expression of lipogenic genes.

**Results:**

Our results showed that the serum levels of ALT, FFAs and TG, as well as the accumulation of hepatic lipid droplets, were increased significantly in mice infected with NS5A-expressing lentiviral particles. NS5A inhibited AMPK phosphorylation and increased the expression levels of sterol regulatory element binding protein-1c (SREBP-1c), acetyl-coenzyme A carboxylase 1 (ACC1) and fatty acid synthase (FASN) in vivo and in vitro. Further investigation revealed that pharmacological activation or ectopic expression of AMPK neutralized the upregulation of SREBP-1c, ACC1 and FASN, and ameliorated hepatic lipid accumulation induced by NS5A. Ectopic expression of SREBP-1c enhanced NS5A-induced hepatic lipid accumulation, which was dramatically reversed by pharmacological activation of AMPK.

**Conclusions:**

Collectively, we demonstrate that NS5A induces hepatic lipid accumulation via the AMPK/SREBP-1c pathway.

## Background

Hepatitis C virus (HCV) infection is a global health problem that has caused a tremendous burden with regard to public health. It is a leading cause of liver diseases including chronic hepatitis, steatosis, fibrosis, cirrhosis and hepatocellular carcinoma [1]. Noticeably, a significant portion of HCV-associated metabolic syndrome complications, such as steatohepatitis, type 2 diabetes mellitus and atherosclerosis, show the association between HCV infection and lipid metabolism dysregulation [2–4]. Hence the precise mechanisms of how HCV regulates hepatic lipid metabolism urgently need to be defined at various molecular levels.

HCV is an enveloped, positive single-stranded RNA (9.6 kb) virus. Its genome encodes a single polyprotein, which is processed by a combination of host and viral proteases to yield 10 individual proteins, including core, envelope 1 and 2 (E1 and E2), p7, and nonstructural proteins (NS2, NS3, NS4A, NS4B, NS5A and NS5B). Nonstructural protein 5A (NS5A) has generated considerable interest in HCV research because of its ability to modulate the virus replication cycle, cell metabolism and host cell interferon response [5–10]. Although recent studies imply that the NS5A protein may play an important role in the pathological changes of HCV-associated hepatic steatosis by interacting with a variety of cellular proteins, the underlying molecular mechanisms remain largely elusive [11–14].

AMP-activated protein kinase (AMPK) is a master metabolic sensor of cellular energy homeostasis and plays critical roles in the regulation of lipid metabolism, including fatty acid synthesis and oxidation [15]. AMPK is a heterotrimeric protein composed of α, β and γ subunits, and its threonine 172 (Thr172) residue phosphorylation at the α subunit has been identified as an essential step in AMPK activation. Activated AMPK augments fatty acid oxidation and impairs lipogenesis by regulating the activities and/or expression levels of transcription factors required for lipogenic genes [10, 16]. Conversely, suppressing AMPKα1 expression level and activity enhances hepatic lipogenesis and lipid accumulation in mice [17]. Indeed, studies indicate that HCV infection reduces the phosphorylation of AMPK at threonine 172 and concomitant AMPK activity, which is partially responsible for HCV-associated hepatic steatosis [18, 19]. However, the role and mechanism of AMPK in NS5A-induced hepatic steatosis are not well understood.

SREBP-1c is a main transcription factor that induces key lipogenic enzymes in the liver [20–22]. Recent studies have shown that HCV alters host lipid metabolism and induces hepatic lipid accumulation by activating of SREBP-1c [23, 24]. Furthermore, our previous study revealed that NS5A upregulates endogenous SREBP-1c by modulating the binding of the transcriptional factor Sp1 to the promoter of SREBP-1c, suggesting that NS5A is a contributing factor in HCV-associated hepatic steatosis [12]. Given the importance of AMPK and SREBP-1c in modulating lipogenic gene expressions, our present study was designed to explore whether NS5A induces hepatic lipid accumulation by modulating the AMPK/SREBP-1c pathway.

In this study, we demonstrated that NS5A increased plasma and hepatic lipid accumulation via the AMPK/SREBP-1c signaling pathway in vivo and in vitro. Our findings highlight the role of NS5A in the development of hepatic steatosis and provide valuable mechanistic insights into HCV-associated hepatic steatosis.

## Methods

### Construction of the lentiviral vectors

The roles of NS5A at the molecular level have been well characterized, but much less is known about the relationship between NS5A and HCV-associated hepatic steatosis. Thus we constructed lentiviral vector for high level and prolonged expression of NS5A. To direct the expression of NS5A to the liver, a chimeric promoter composed of the mouse alpha-fetoprotein enhancer II (AFP) and the minimal mouse albumin promoter was instrumental. First, the pLive-lacZ vector, which contains a chimeric promoter composed of a mouse alpha-fetoprotein enhancer II, a minimal mouse albumin promoter and a multiple cloning site (MCS) between two introns, was digested with PacI and BglII and subsequently amplified by PCR using primers incorporating the restriction site (sense: 5′-TAAGGCCTACGCGTA-3′; antisense: 5′-GATCTACGCGTAGGCCTTAAT-3′, the underlined nucleotides are the restriction site for MluI) to generate a modified pLive-lacZ vector. Then the pTRIP and modified pLive-lacZ vectors were digested with MluI and NheI, and a chimeric promoter composed of a mouse alpha-fetoprotein enhancer II, a minimal mouse albumin promoter and an intron was ligated into the pTRIP vector. The full coding sequences of NS5A and enhanced green fluorescent protein (EGFP) were amplified by PCR and cloned into the modified pTRIP vector at the NheI/XhoI sites. The generated recombinant lentiviral expression vectors were verified by sequence determination and termed pTRIP-NS5A and pTRIP-EGFP respectively.

### Cell culture

The human embryonic kidney cell line HEK293T, mouse hepatoma cell line Hepa1–6 and human hepatoma cell line HepG2 were maintained in Dulbecco’s modified Eagle’s medium (Gibco, CA, USA) supplemented with 10% fetal bovine serum (Gibco, CA, USA), 100 U/mL penicillin and 100 μg/mL streptomycin at 5% CO_2_ and 37 °C.

### Packaging and identification of lentivirus particles

Lentiviral particles were prepared using the GM easy™ lentiviral packaging kit (Genomeditech, Shanghai, China). In brief, HEK293T cells were seeded at a density of 2 × 10^6^ in a 10-cm cell culture dish containing 10 ml of media. The next day, the cells were co-transfected with a complex containing the recombinant lentiviral construct expressing NS5A or EGFP (pTRIP-NS5A or pTRIP-EGFP), and three helper plasmids (GAG/Pol, REV and VSV-G from the kit) according to the manufacturer’s instructions. The medium was changed 18–20 h after transfection. The supernatant was collected after an additional 48 h. The viral particles were concentrated and stored at − 80 °C until use. The titer of the viral particles was determined by quantitative real-time PCR (the primers are listed in Table 1).

**Table 1:** Primers used to detect gene expressions at mRNA level

Gene name	Primer sequences (5′-3′)
NS5A	F: 5′-ATTGGCTGCGTGACATCTG-3’
	R: 5′-ACCACGCTCTGCTCCTCACT-3’
Mouse SREBP-1c	F: 5′-GGAGCCATGGATTGCACATT-3’
	R: 5′-GCTTCCAGAGAGGAGCCCAG-3’
Mouse FASN	F: 5′-CACAGATGATGACAGGAGATGGA-3’
	R: 5′-TCGGAGTGAGGCTGGGTTGATA-3’
Mouse ACC1	F: 5′-TGTTGGGGTTATTTCAGTGTTGC-3’
	R: 5′-TGTCCAGCCAGCCAGTGTCG-3’
Mouse β-Actin	F: 5′-TTCCTTCTTGGGTATGGAAT-3’
	R: 5′-GAGCAATGATCTTGATCTTC-3’

*F* Forward primer, *R* Reverse primer

### Animals and treatments

Male C57BL/6 J wild-type mice (9 weeks old, body weight 21~26 g, purchased from HFK Bioscience Co., LTD, Beijing, China) were bred and housed under a 12/12 h light/dark cycle with free access to normal diet and water in specific pathogen-free conditions at the Tianjin Medical University Animal Center. The mice were divided into three groups and each group comprised 12 animals. To deliver the viral particles, the experimental groups were injected with the recombinant lentiviral particles (2.0 × 10^7^ TU/100 μl/mouse) expressing NS5A or EGFP via the tail vein once a week for 3 weeks. The groups injected with the EGFP lentiviral particles or normal saline were used as controls. Three days after the final injection, the mice were fasted overnight and humanely sacrificed. Blood and liver tissue samples were collected for analyses. All the experiments involving animals were conducted in accordance with the Chinese guidelines for animal welfare and experimental protocol, which was approved by the Animal Care and Use Committee of Tianjin Medical University.

### Serum assays

The mouse serum ALT levels were measured by the Reitman-Frankel method according to the manufacturer’s protocols (Rong Sheng, Shanghai, China)**.** The mouse serum FFAs levels were measured using a chemical colorimetry assay with a non-esterified FFA assay kit, and the mouse serum TG levels were determined using an enzymatic colorimetric method with a triglyceride reagent kit according to the manufacturer’s instructions (Jiancheng Bioengineering Institute, Nanjing, China).

### Hematoxylin and eosin (H&E) staining

Five micrometer-thick sections were cut from each frozen liver specimen. For histopathologic examination under a light microscope, the slides were first incubated with hematoxylin for 30–60 s and then washed with 1% ethanol hydrochloride for 3 s. After washing with water, the slides were stained with eosin for 30–60 s and subsequently dehydrated with graded dilutions of ethanol. Each section was assessed according to 10 × 40 light microscopic fields, and the vacuoles in the cytoplasm were considered as lipid droplets [25]. The severity of steatosis was scored according to the criteria in a previous study [26].

### Oil red O staining

Frozen liver tissues were cut into 5-μm sections and affixed to microscope slides. HepG2 cells were seeded in a 12-well plate containing a glass coverslip bottom. The cells attached to the coverslip were fixed in 4% paraformaldehyde for 15 min. The liver sections and HepG2 cells were analyzed with an Oil Red O staining kit (Jiancheng Bioengineering Institute, Nanjing, China) according to the supplier’s instructions. The lipid droplets stained with Oil Red O were visualized with an Olympus BX53 microscope (Olympus Corporation, Tokyo, Japan) equipped with a DP72 Microscope Digital Camera and Image-Pro Plus 7.0 software [27]. Light absorbance of the extracted dye was measured at 520 nm.

### Immunohistochemistry (IHC) staining

The expression levels of NS5A, SREBP-1 and phospho-AMPKα (Thr172) in liver samples were measured using IHC staining. In brief, specimens were fixed in 4% paraformaldehyde overnight and then embedded in paraffin wax according to standard methods. Following antigen retrieval by heating the slices in a microwave for 30 min, the deparaffinized liver sections were incubated with a 3% H_2_O_2_ solution for 30 min to quench endogenous peroxidase activity. The slides were incubated overnight at 4 °C with anti-phospho-AMPKα (Thr172) (Affinity, OH, USA), anti-NS5A or anti-SREBP-1 (Abcam, Cambridge, UK). Negative controls were obtained by omitting the primary antibody and using primary antibody diluent. After washing, the slides were incubated with anti-rabbit or mouse Plus-HRP (ZSJQ-BIO, Beijing, China) for 1 h at room temperature. Then, the sections were incubated with N, N-dimethylaminoazobenzene (DAB) (ZSJQ-BIO, Beijing, China) and the nuclei were counterstained with hematoxylin (ZSJQ-BIO, Beijing, China). The images were captured using an Olympus BX53 microscope at × 400 or × 1000 magnification.

### Quantitative real-time PCR (qRT-PCR)

Total RNA was extracted using the E.Z.N.A.® HP Total RNA Extraction Kit (Omega Bio-Tek, GA, USA) and then reverse-transcribed to cDNA using the ReverAid First Strand cDNA Synthesis Kit (Fermentas Inc., Canada). The expression levels of *SREBP-1c, FASN* and *ACC1* were detected using a SYBR green real-time PCR kit according to the manufacturer’s instructions (Sangon Biotech, Shanghai, China). The primer sequences are listed in Table 1. The expression levels of above genes were normalized to those of β-Actin and measured by the comparative Ct (2^−ΔΔCt^) method.

### Western blotting

Tissues and cells were lysed, and identical amounts of proteins (20 μg/lane) were separated by SDS-PAGE and transferred onto nitrocellulose membranes. The membranes were incubated overnight with the following primary antibodies: anti-NS5A, anti-SREBP-1c (Abcam, Cambridge, UK), anti-AMPKα1, anti-AMPKα2, anti-FASN, anti-ACC1 (Proteintech, Wuhan, China), anti-Flag (Sigma-Aldrich, St. Louis, USA), anti-phospho-AMPKα (Thr172) (Affinity, OH, USA), or anti-β-Actin (RayBiotech, Beijing, China). Detection was performed using the corresponding horseradish peroxide-labeled IgG (ZSJQ-Bio, Beijing, China) followed by chemiluminescence (Advansta, CA, USA).

### Cell transfection and incubation with pharmacological compounds

In brief, Hepa1–6 and HepG2 cells seeded in a 6-well or 12-well plate were transfected using Lipofectamine® 3000 transfection reagent (Invitrogen, CA, USA) according to the manufacturer’s protocol. The following plasmids were used: pCMV-Flag-NS5A, pcDNA3.1-2xFlag-SREBP-1c and pcDNA3.1 AMPKα1 and α2 (from Addgene). AMPK activator 5-aminoimidazole-4-carboxamide-1-β-D-ribofuranoside (AICAR) was added when indicated [28]. After transfection and/or pharmacological intervention as indicated in the figure, the cells were subjected to qRT-PCR, Western blotting and Oil Red O staining.

### Data analysis

The data are expressed as mean ± *S.D*. Statistical comparisons between two groups were made using Student’s t-test. One-way ANOVA was used for the bar graphs containing three or more groups. *P < 0.05, **P < 0.01 and ***P < 0.001 indicate significant differences, and a non-significant difference is noted by NS*.*

## Results

### NS5A induced lipid metabolic alterations in vivo

**Fig. 1:**
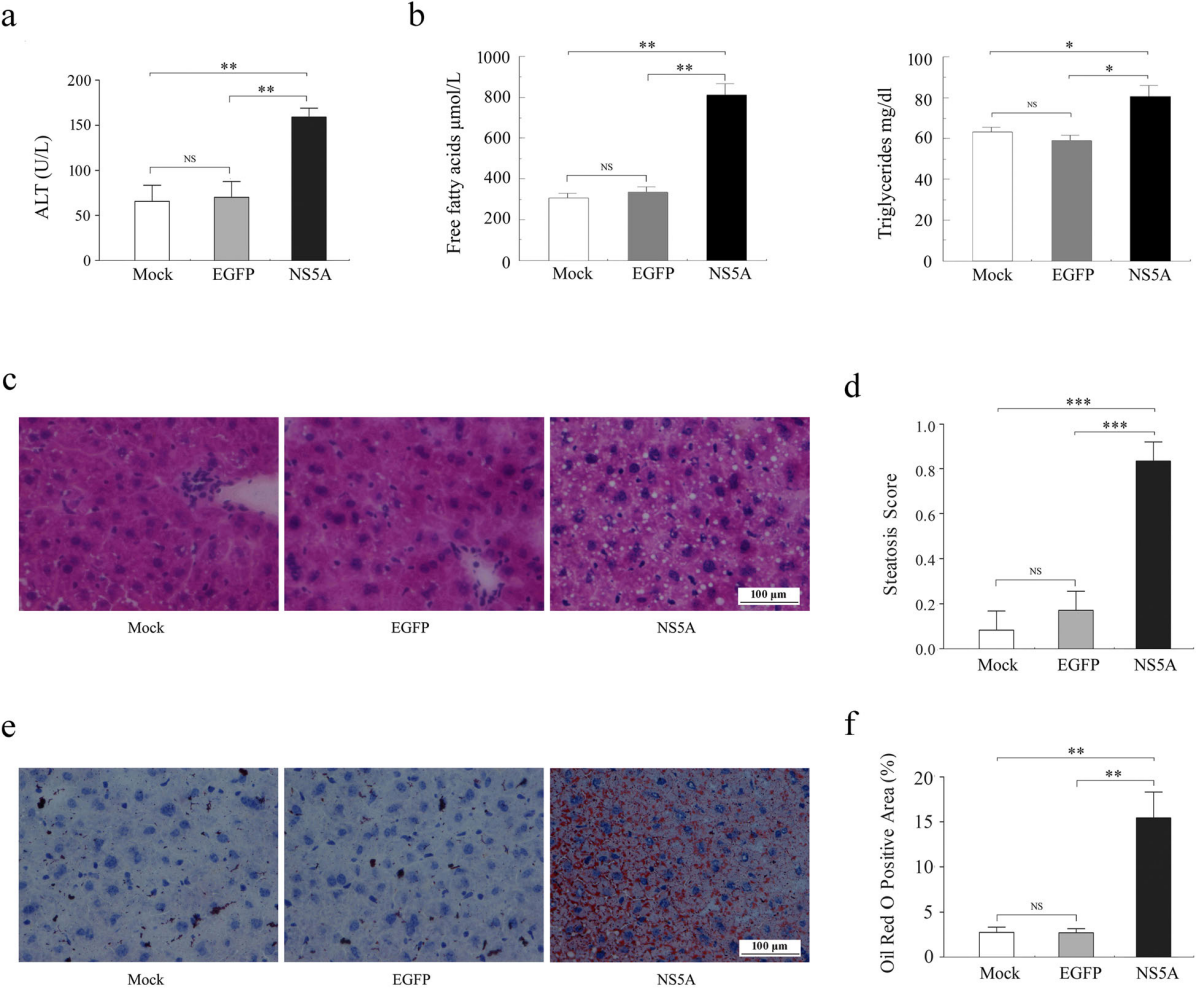
NS5A induces lipid metabolic alterations in vivo*.* **a** Serum levels of ALT in the Mock group (white bar), EGFP group (gray bar), and NS5A group (black bar). **b** Serum levels of FFAs (left) and TG (right) in C57BL/6 J mice were assessed. **c** Representative H&E staining of frozen liver sections from mice treated with normal saline or recombinant lentiviral particles expressing NS5A or EGFP. **d** H&E-stained liver sections were evaluated for steatosis and scored as described in methods sections. **e** and **f** Effects of NS5A on lipid storage as detected by Oil Red O staining. NS5A-expressing mice showed massive lipid droplets in their liver tissues compared with EGFP-expressing mice or mice treated with normal saline. Mock: treatment with normal saline; EGFP: treatment with EGFP-expressing lentiviral particles; NS5A: treatment with NS5A-expressing lentiviral particles. H&E, hematoxylin and eosin. Original magnification: × 400, Scale bar = 100 μm; *n* = 12 in each group. Statistically significant differences are indicated: NS, no significance, *P < 0.05, **P < 0.01, ***P <0.001

To investigate whether the NS5A protein is involved in HCV-associated hepatic steatosis in vivo, we generated model mice by injecting recombinant lentiviral particles expressing the NS5A protein via the tail vein. The serum levels of ALT, FFAs and TG were examined. No significant differences in the serum levels of ALT, FFAs and TG were observed between the EGFP group and the Mock group. Comparison of the NS5A group and each control group revealed significantly increased serum levels of ALT, FFAs and TG in NS5A-expressing mice (Fig. 1a, b). Furthermore, the histological alterations in liver tissues were extensively examined by H&E and Oil Red O staining. H&E staining showed massive cytoplasmic vacuoles (Fig. 1c, d), and Oil Red O staining showed significantly enhanced lipid droplets accumulation in the hepatocytes of mice expressing NS5A compared to those in either of the control groups (Fig. 1e, f). Among the 12 NS5A-expressing mice, 2 (16.67%) had no hepatic steatosis (score 0), and 10 (83.33%) mice had mild or moderate (score 1) or severe (score 2) hepatic steatosis. Our results suggest that NS5A induces dyslipidemia and considerable hepatic steatosis in vivo.

### NS5A regulates the expression of lipogenic genes in vivo

**Fig. 2:**
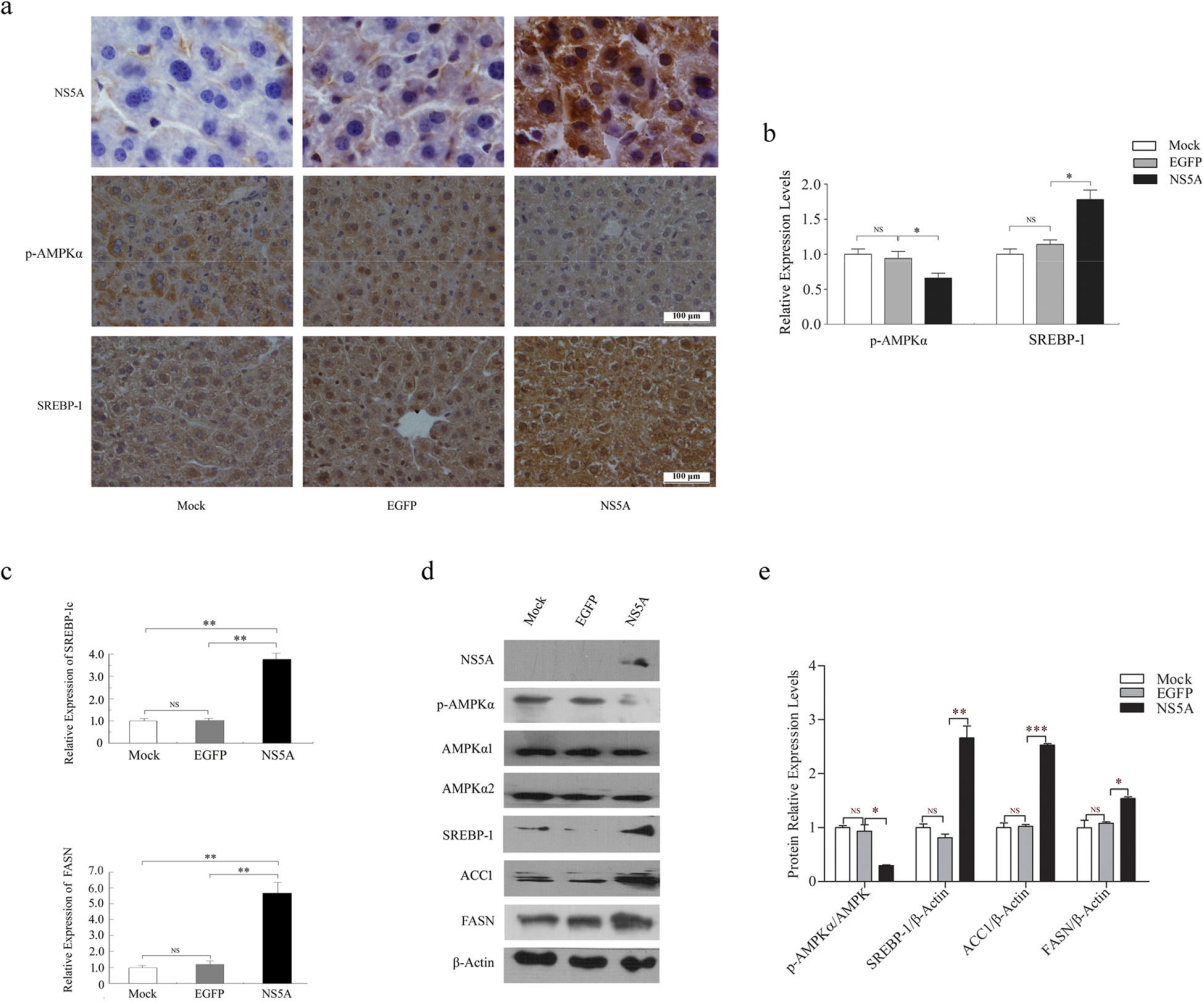
NS5A regulates the expression of lipogenic genes in vivo*.* **a** Representative IHC staining for NS5A, p-AMPKα and SREBP-1. *n* = 5 animals per group. Original magnification: × 400 (p-AMPKα, SREBP-1), × 1000 (NASA). Scale bar = 100 μm. IHC, immunohistochemistry. **b** IHC staining intensity and area were evaluated using a semi-quantitative scoring system. **c** The expression levels of *SREBP-1c* and *FASN* were examined by qRT-PCR in mixed liver tissues from each group. The mRNA levels were normalized to β-Actin and relative fold changes in expression levels were compared with those of the wild-type mice in the Mock group, in which the expression was set to 1.0 (*n* = 5 animals per group; **P < 0.01 in the NS5A group vs. Mock or EGFP group). **d** The levels of p-AMPKα, SREBP-1, FASN and ACC1 in liver tissues were analyzed by Western blotting. β-Actin was used as a loading control. **e** The fold-change was calculated based on a densitometric analysis of the band intensities. Statistically significant differences are indicated: NS (no significance), *P < 0.05, **P < 0.01, ***P < 0.001

As described, both AMPK and SREBP-1c are key regulators of lipid metabolism. AMPK is almost universally expressed in eukaryotic cells and activated-AMPK inhibits lipogenesis, but SREBP-1c is particularly abundant in the liver and its actions are focused on lipid synthesis. It has been reported that the overexpression of SREBP-1c and its target genes is involved in HCV-associated hepatic steatosis. In addition, a study has indicated that the phosphorylation of AMPKα at Thr172 (in short, p-AMPKα) and concomitant AMPK activity are dramatically reduced in cells infected with HCV [18]. Thus, we presumed that AMPK and SREBP-1c are strong candidates to mediate NS5A-induced hepatic lipid accumulation. Serial sections of liver tissues were prepared and stained simultaneously. IHC staining showed that NS5A was widely expressed in the liver tissues of the NS5A group but not in those of the control EGFP and Mock groups (Fig. 2a). To explore the roles of AMPK/SREBP-1c in NS5A-induced hepatic steatosis, we examined the expression of p-AMPKα and SREBP-1 in mouse liver tissues by IHC staining. For semi-quantitative analysis of IHC staining, at least five randomly chosen microscopic visual fields of a sample from each group were counted. Our results showed strong p-AMPKα staining in the Mock and EGFP groups and weak staining in the NS5A group. In contrast, the expression of SREBP-1 in the NS5A group was significantly higher than that in the Mock or EGFP group (Fig. 2a, b). To gain insight into the mechanism by which NS5A induces hepatic lipid accumulation, we further explored the lipogenic gene expression responsible for the rate-limiting steps of fatty acid synthesis in the liver tissues of NS5A-expressing mice. Quantitative real-time PCR (qRT-PCR) analysis demonstrated that the transcriptional levels of *SREBP-1c* and *FASN* were significantly higher in the liver tissues of the NS5A group than in those of each control group, suggesting that NS5A promotes the expression of both *SREBP-1c* and *FASN* at the mRNA level (Fig. 2c). Western blotting analysis also validated that NS5A decreased p-AMPKα with no change in the total level of AMPK and increased the expression levels of SREBP-1, FASN and ACC1 (Fig. 2d, e). Together, our results indicate that NS5A decreases the Thr172 phosphorylated form of AMPK and significantly increases the mRNA and protein expression levels of *SREBP-1c* and its target genes in vivo.

### NS5A disrupts lipid metabolism and induces hepatic lipid accumulation in vitro

**Fig. 3:**
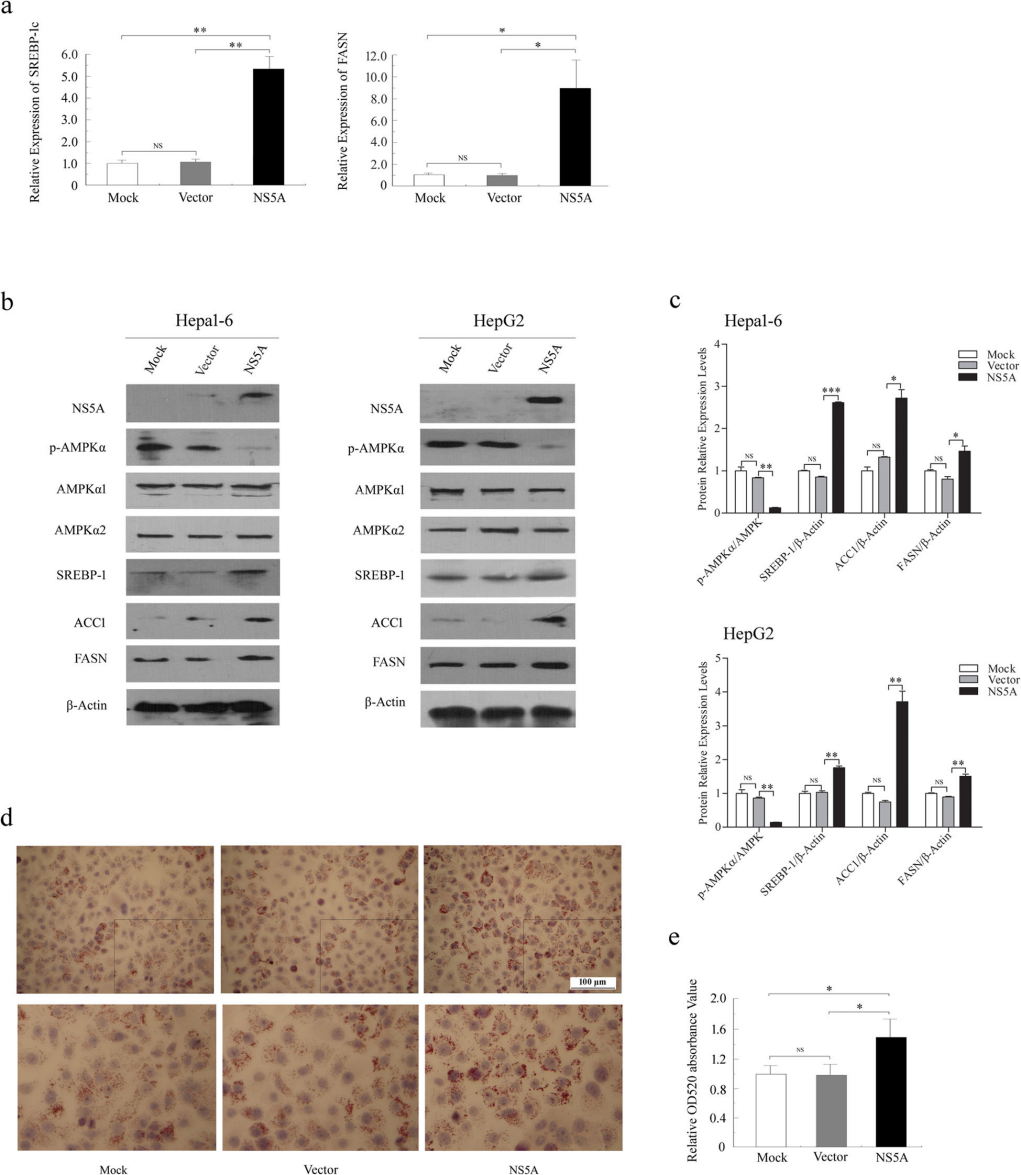
NS5A disrupts lipid metabolism and induces hepatic steatosis in vitro*.* **a** The mRNA expression levels of *SREBP-1c* and *FASN* in Hepa1–6 cells were quantified by real-time PCR and relative expression values were normalized to those of β-Actin. **b** Western blotting analysis of lipogenic gene expression in NS5A-expressing Hepa1–6 and HepG2 cells. **c** Levels of p-AMPKα, SREBP-1, FASN and ACC1 were quantified by densitometry and compared with those of AMPK1/2 or β-Actin. **d** Representative images of lipid accumulation in NS5A-transfected HepG2 cells with Oil Red O staining. **e** Oil Red O extracted with isopropanol was measured at OD520. The values represent the mean ± *SD* of three experiments. Mock: non-transfected cells; Vector: the cells transfected with empty plasmid; NS5A: the cells transfected with NS5A expressing plasmid. NS (no significance), *P < 0.05, **P < 0.01, ***P < 0.001

To determine whether these in vivo findings can be demonstrated in vitro*,* we explored the effects of NS5A on hepatic lipid accumulation by transiently transfecting NS5A-expressing plasmids in mouse hepatoma cell Hepa1–6 and human hepatoma cell HepG2 cells. The qRT-PCR analysis revealed that the relative mRNA expression levels of *SREBP-1c* and *FASN* were much higher in NS5A-transfected Hepa1–6 cells (in short, NS5A) than in non-transfected (in short, Mock) or empty vector-transfected cells (in short, Vector) (Fig. 3a). Moreover, Western blotting analysis showed that p-AMPKα levels were attenuated, whereas SREBP-1, ACC1 and FASN levels were increased in the NS5A group (Fig. 3b, c). Compared with the Mock or Vector conditions, NS5A caused obvious induction of lipid deposition in HepG2 cells, as evidenced by Oil Red O staining (48.3% increase in Oil Red O staining compared to that in the control transfection conditions) (Fig. 3d, e). These results are consistent with our observations in mice, suggesting that AMPK and SREBP-1c play important roles in NS5A-induced lipid accumulation both in vivo and in vitro.

### AICAR reverses the effects of NS5A on hepatic lipid metabolism via activation of AMPK

**Fig. 4:**
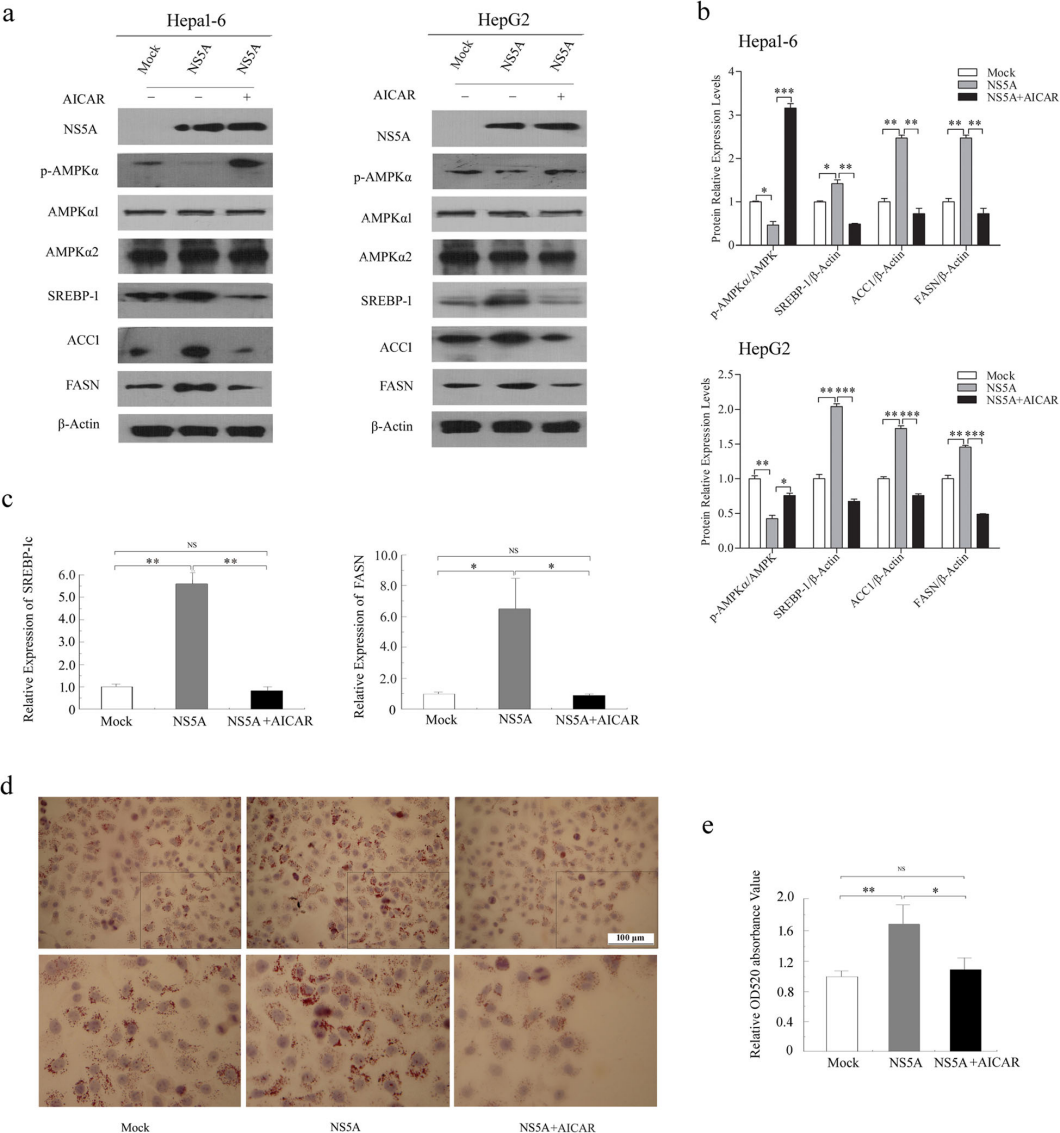
AICAR reverses the effects of NS5A on hepatic lipid metabolism via activation of AMPK. **a** and **b** The effects of AICAR on the expression of lipogenic genes in Hepa1–6 and HepG2 cells were measured using Western blotting. Hepa1–6 and HepG2 were transfected with NS5A and then treated with AICAR for 8–10 h. **c** AICAR inhibited *SREBP-1c* and *FASN* mRNA expression in Hepa1–6 cells as measured by qRT-PCR. **d** and **e** Representative microscopic images of Oil Red O staining of HepG2 cells transfected with a NS5A-expressing plasmid following treatment with AICAR (1 mM) for 8–10 h. Oil Red O extracted with isopropanol was measured at OD520. The values represent the mean ± *SD* of three experiments. NS (no significance), *P < 0.05, **P < 0.01, ***P < 0.001

To confirm that phosphorylation and activation of AMPK are inhibited by NS5A during lipid accumulation, an AMPK agonist, aminoimidazole carboxamide ribonucleotide (AICAR), was used to stimulate hepatoma cells after transfection with the NS5A-expressing plasmid. Hepa1–6 and HepG2 cells were transfected with NS5A for 40 h and then stimulated with AICAR (1 mmol/L) for another 8–12 h. Our results showed that activation of AMPK by AICAR completely abrogated the inhibition of AMPK phosphorylation and increased the protein expression levels of SREBP-1c and its downstream genes (Fig. 4a, b). In addition, AMPK activation via AICAR inhibited the mRNA expression of *SREBP-1c* and *FASN* in Hepa1–6 cells transfected with the NS5A-expressing plasmid compared with the empty vector transfection conditions (Fig. 4c). More importantly, administration of AICAR largely ablated NS5A’s effects on lipid accumulation in HepG2 cells, as evidenced by Oil Red O staining (Fig. 4d, e). Together, the AMPK agonist AICAR reverts lipid metabolism disorders by increasing AMPK Thr172 phosphorylation and decreasing the expression levels of SREBP-1c, ACC1 and FASN, which results in the significant improvement of NS5A-induced lipid accumulation in hepatocytes.

### Ectopic expression of AMPKα attenuates the role of NS5A in lipogenesis

**Fig. 5:**
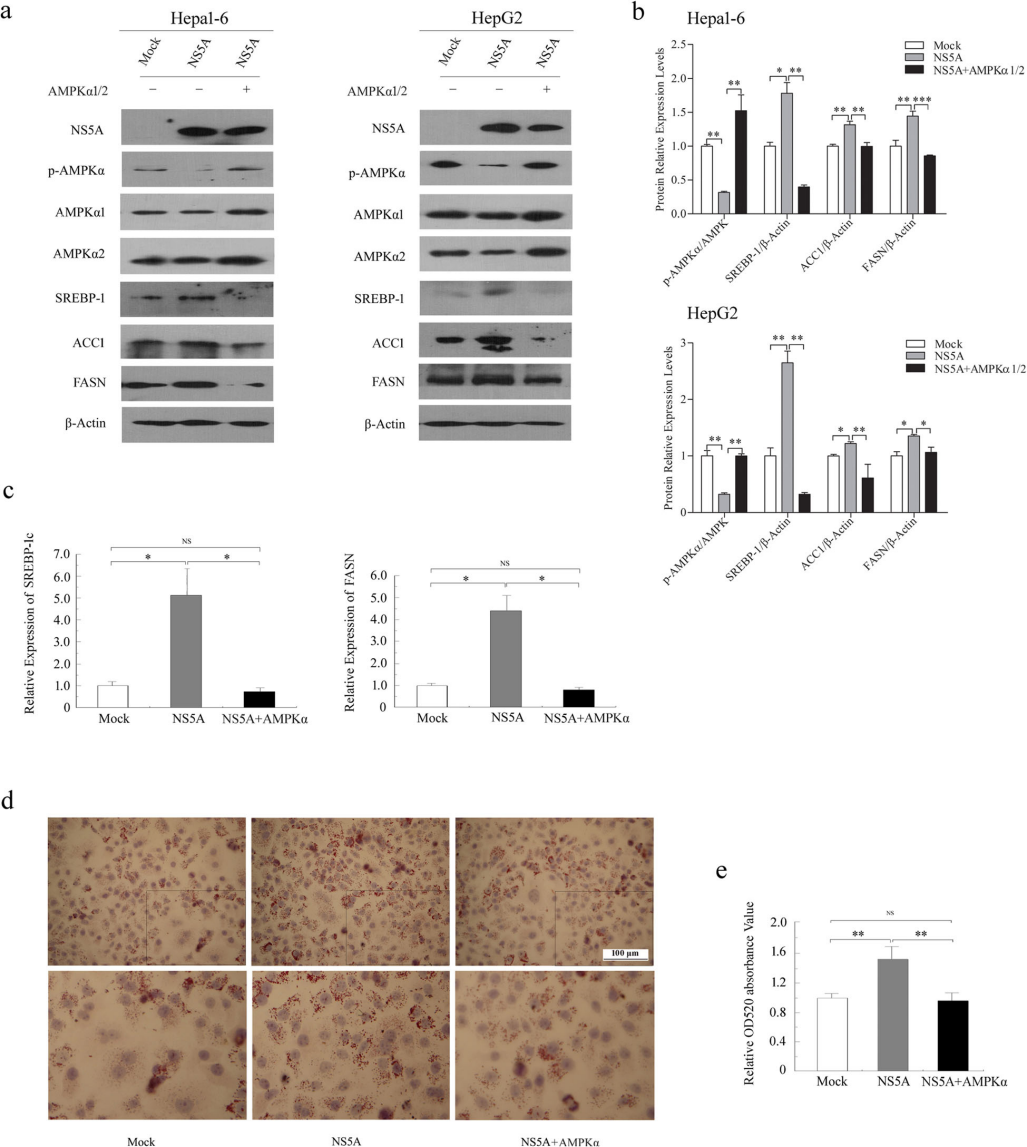
Ectopic expression of AMPKα attenuates the role of NS5A in lipogenesis. (**a** and **b**) The levels of p-AMPKα, SREBP-1, FASN and ACC1 were measured in hepatocellular carcinoma Hepa1–6 and HepG2 cells co-transfected with NS5A and AMPKα1 and 2 by Western blotting. (**c**) The mRNA levels of *SREBP-1c* and *FASN* in Hepa1–6 cells were examined using qRT-PCR. (**d** and **e**) Representative images of the accumulation of cytoplasmic lipid droplets in HepG2 cells co-transfected with NS5A and AMPKα1 and 2 for 40 h stained with Oil Red O (× 400). Oil Red O extracted with isopropanol was measured at OD520. The Data are representative of three independent experiments. NS (no significance), *P < 0.05, **P < 0.01, ***P < 0.001

To further confirm our results and understand whether increased p-AMPK attenuates NS5A-induced lipogenesis, Hepa1–6 and HepG2 cells were co-transfected with NS5A-, AMPKα1- and AMPKα2-expressing plasmids. As expected, our results showed that additional expression of AMPKα1 and 2 increased the level of AMPK Thr172 phosphorylation, thus neutralizing the inhibition of AMPK activity and upregulation of SREBP-1c, ACC1 and FASN induced by NS5A (Fig. 5a, b). The qRT-PCR analysis validated that the relative mRNA expression levels of *SREBP-1c* and *FASN* were decreased in Hepa1–6 cells after co-transfection with NS5A and AMPKα1 and 2 (Fig. 5c). As a result, over-expressing AMPKα1 and α2 rescued the NS5A-induced lipid accumulation observed by Oil Red O staining (Fig. 5d, e). These data indicate that increased levels of AMPK Thr172 phosphorylation improve the lipid metabolism disorder induced by NS5A. Combined, we confirm that inhibition of AMPK phosphorylation at Thr172 is required for NS5A to induce lipid accumulation.

### Paradoxical roles of AMPK and SREBP-1c in NS5A-induced hepatic steatosis

**Fig. 6:**
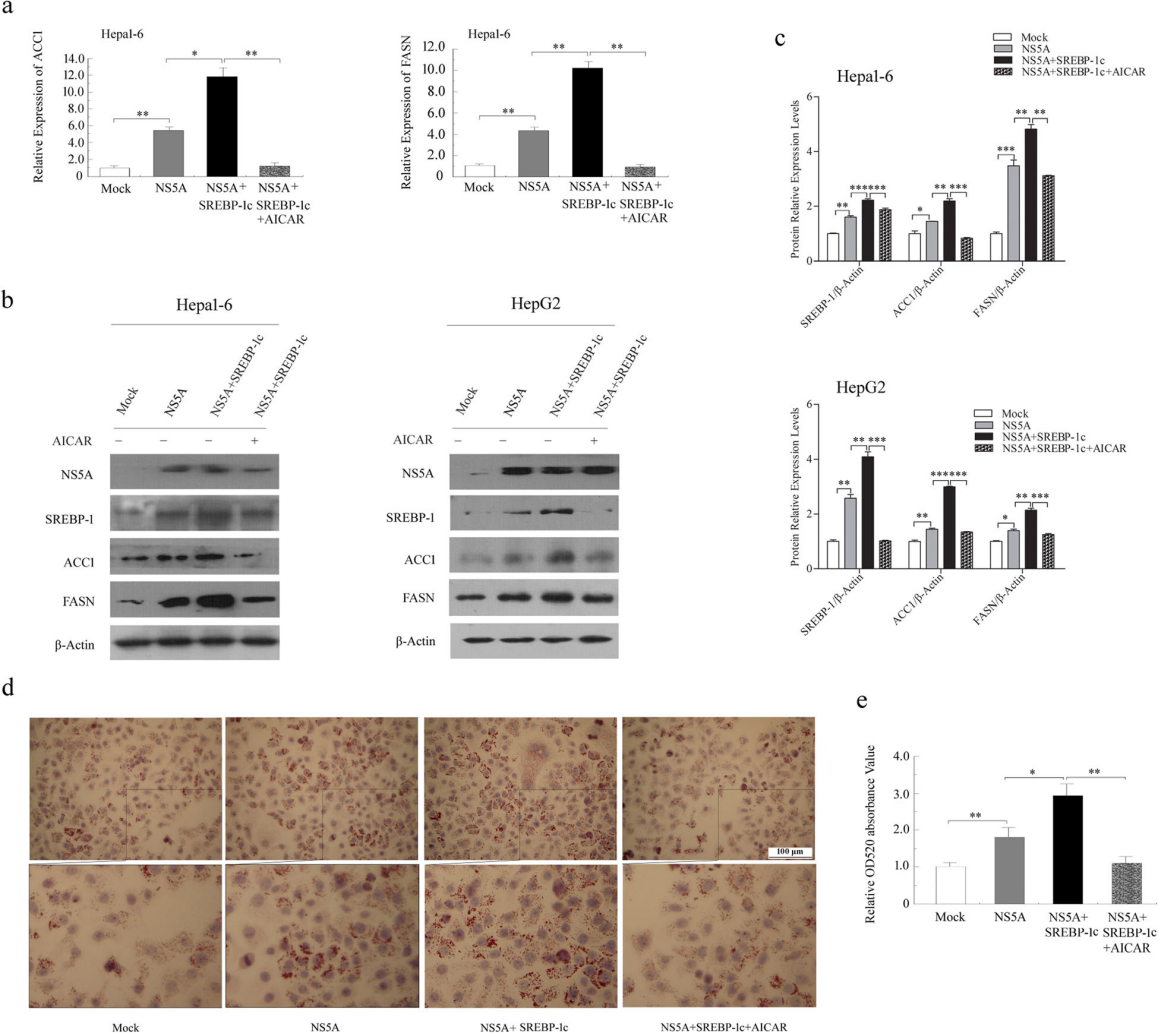
Paradoxical roles of AMPK and SREBP-1c in NS5A-induced hepatic steatosis. **a** qRT-PCR analysis of the mRNA levels of *FASN* and *ACC1* in Hepa1–6 cells co-transfected with NS5A and SREBP-1c. **b** and **c** The expression levels of SREBP-1c, ACC1 and FASN were analyzed by Western blotting in Hepa1–6 and HepG2 cells after co-transfection with NS5A and SREBP-1c. **d** and **e** The accumulation of lipid droplets in HepG2 cells was visualized by Oil Red O staining. Oil Red O extracted with isopropanol was measured at OD520. The Data are representative of three independent experiments. NS (no significance), *P < 0.05, **P < 0.01, ***P < 0.001

To definitively determine the roles of AMPK and SREBP-1c in NS5A-induced steatosis, Hepa1–6 and HepG2 cells were co-transfected with NS5A and SREBP-1c for 40 h and then treated with 1 mmol/L AICAR for another 8–12 h before qRT-PCR, Western blotting and Oil Red O staining assays. Our results showed that the relative mRNA expression levels of *FASN* and *ACC1* were further increased in Hepa1–6 cells co-transfected with NS5A and SREBP-1c compared to those in empty vector or NS5A-transfected cells, but these changes were reversed in the presence of AICAR (Fig. 6a). It was also noteworthy that increased protein levels of FASN and ACC1 were all substantially reversed by treatment with AICAR in co-transfected cells (Fig. 6b, c). Accordingly, overexpression of SREBP-1c further enhanced lipid accumulation in co-transfected cells compared to NS5A transfection. Moreover, AMPK activation by AICAR ameliorated the synergistic effects of NS5A and SREBP-1c on hepatic lipid accumulation in co-transfected HepG2 cells, as evidenced by a significant reduction in Oil Red O staining (Fig. 6d, e). These results further demonstrate that SREBP-1c promotes NS5A-induced hepatic steatosis. Moreover, activation of AMPK by AICAR inhibited SREBP-1c and its target gene expression levels and protected against NS5A-induced hepatic steatosis. Taken together, it seems clear that NS5A enhances plasma and hepatic lipid accumulation by suppressing AMPK phosphorylation and activation and consequently increasing the expression levels of SREBP-1c and its target genes in vivo and in vitro.

Over all, we conclude that NS5A modulated the expression of lipogenic genes and led to hepatic lipid accumulation, in which AMPK was implicated as a possible regulator.

## Discussion

Lipid homeostasis plays a crucial role in maintaining normal physiological function, and its dysregulation results in the development of obesity, diabetes, cardiovascular disease and cancer. HCV appears to be closely connected to host-cell lipid metabolism. On the one hand, the intracellular lipid levels and fatty acid composition have a strong effect on the HCV life cycle. HCV replication complex and HCV virion assembly and release are tightly linked to host cell lipoproteins and lipids [10, 29]. On the other hand, HCV proteins modulate host-cell lipid metabolic processes in many ways, including membrane biogenesis, lipid droplet formation and lipid trafficking [30–32].

Steatosis is a frequent histological feature of HCV infection. Numerous cellular and viral proteins have been implicated in the modulation of hepatic steatosis. The HCV NS5A protein, a master regulator of HCV replication and assembly, has been found to colocalize with apolipoprotein A1 around the lipid droplets of steatotic hepatocytes [33]. NS5A affects the formation of lipid droplets and alters lipid droplet morphology and size [34]. NS5A augments the transcriptional activity and gene expression of PPARgamma, and induces lipid accumulation in hepatic cells [32]. Although NS5A may play a vital role in this process, the underlying mechanisms remain obscure. To better understand the significance of NS5A in hepatic steatosis, we generated a mouse model by intravenously injecting recombinant lentiviral particles carrying the coding region of the NS5A protein, under the control of a mouse albumin promoter and AFP enhancer, into C57BL/6 J mice via their tail vein. We found that the serum levels of FFAs and TG and the abundance of hepatic lipid droplets were increased significantly in NS5A-infected mice compared with those in Mock (mice injected with normal saline) or EGFP mice (mice infected with EGFP-encoding lentiviral particles). These findings offer valuable evidence that NS5A might play an etiological role in HCV-associated hepatic steatosis.

To identify the mechanism of hepatic lipid metabolism disorders mediated by NS5A, we further explored the expression of lipogenic genes. AMPK, a major cellular energy sensor, plays a critical role in lipid homeostasis. Critically, it has been reported that phosphorylation of AMPKα at residue Thr172 (p-AMPK) is decreased in HCV-infected cells, indicating that AMPK participates in the pathogenesis of HCV-associated hepatic steatosis [18, 35]. Therefore, we hypothesized that NS5A mediates hepatic steatosis by inhibiting AMPK activation. SREBPs are a family of transcription factors that regulate lipid homeostasis by controlling the expression of a range of enzymes required for endogenous cholesterol, fatty acid, triacylglycerol and phospholipid synthesis. It has been reported that HCV promotes the accumulation of intracellular lipids through enhancing de novo lipogenesis by activating SREBPs [24]. Moreover, our previous study has indicated that NS5A activates SREBP-1c transcription and increases the level of endogenous SREBP-1 [12]. Hence, we focused on investigating the AMPK/SREBP-1c pathway and its downstream genes involved in lipogenesis. We found that phosphorylation of AMPKα (Thr172) was decreased and that SREBP-1c, ACC1 and FASN mRNA and/or protein levels were increased significantly in mouse hepatic tissues and hepatoma cell lines expressing NS5A. Our results suggested that NS5A-induced high expression of these lipid synthesis-related genes might be related to the AMPK/SREBP-1c pathway.

AMPK reportedly plays a pivotal role in many cellular signaling cascades [36–39]. To this end, we investigated whether NS5A mediates the AMPK/SREBP-1c pathway to disrupt lipid homeostasis. Hepa1–6 and HepG2 cells were transfected with NS5A followed by treatment with AICAR, an AMPK activator. Noticeably, increased p-AMPK by AICAR attenuated the levels of SREBP-1c, ACC1 and FASN and simultaneously reduced lipid droplet abundance in the presence of NS5A. To further confirm the role of the AMPK/SREBP-1c pathway, Hepa1–6 and HepG2 cells were co-transfected with NS5A, AMPKα1 and AMPKα2. Similar results were observed that increased p-AMPK and decreased SREBP-1c levels diminished the role of NS5A in hepatic lipid accumulation. In the absence or presence of the AICAR compound, we further examined the opposing roles of SREBP-1c and AMPK in lipid metabolism in Hepa1–6 and HepG2 cells co-transfected with NS5A- and SREBP-1c-expressing plasmids. Consistent with our hypothesis, ectopic expression of SREBP-1c enhanced lipid accumulation in N5SA-expressing cells, and pharmacological intervention with AICAR alleviated the levels of SREBP-1c and its downstream genes and subsequently led to decreased lipid droplet formation. Collectively, our research revealed that NS5A inhibits the phosphorylation of AMPK at Thr172, and augments the expression of SREBP-1c and its targeted genes, resulting in hepatic steatosis.

## Conclusions

In conclusion, our results further elucidated the molecular mechanisms of HCV-associated hepatic steatosis by demonstrating the important role of the AMPK/SREBP-1c pathway in NS5A-induced hepatic steatosis. New light is shed by the identification of NS5A-mediated AMPK phosphorylation and activation as a signaling hub capable of contributing to HCV-associated hepatic steatosis. Hence, the AMPK/SREBP-1c pathway may be a potential therapeutic target for the treatment of NS5A-induced hepatic steatosis.

## Data Availability

The authors confirm that all materials described in the manuscript are fully available to any scientist wishing to use them, without restriction.

## Abbreviations

ACC1: Acetyl-coenzyme A carboxylase 1
ALT: Alanine aminotransferase
AMPK: AMP-activated protein kinase
EGFP: Green fluorescent protein
FASN: Fatty acid synthase
FFA: Free fatty acids
H&E: Hematoxylin and eosin
HCV: Hepatitis c virus
NS5A: Nonstructural protein 5A
qRT-PCR: Quantitative real-time PCR
SREBP-1c: Sterol regulatory element-binding protein-1c
TG: Triglycerides
